# Relationship Between Short-Term Postural Responses to Noisy Galvanic Vestibular Stimulation at Varying Current Intensities and Its Prolonged Effects in Patients With Peripheral Vestibulopathy

**DOI:** 10.7759/cureus.95861

**Published:** 2025-10-31

**Authors:** Chisato Fujimoto, Takuya Kawahara, Yayoi S Kikkawa, Makoto Kinoshita, Teru Kamogashira, Mineko Oka, Kentaro Ichijo, Kenji Kondo, Shinichi Iwasaki

**Affiliations:** 1 Otorhinolaryngology and Head and Neck Surgery, The University of Tokyo, Bunkyo, JPN; 2 Otolaryngology, Tokyo Teishin Hospital, Chiyoda, JPN; 3 Clinical Research Promotion Center, The University of Tokyo Hospital, Bunkyo, JPN; 4 Otolaryngology - Head and Neck Surgery, Nagoya City University, Nagoya, JPN

**Keywords:** electric stimulation, posture, vestibular diseases, vestibular system, vestibule

## Abstract

Objectives: For patients with peripheral vestibulopathy, the optimal intensity of noisy galvanic vestibular stimulation (nGVS) for improving postural stability can vary from day to day, yet frequent measurement is clinically challenging. This study aimed to investigate the relationship between the short-term effects of nGVS at various current intensities on postural stability and the prolonged effects of nGVS applied at the optimal intensity on different days. Specifically, we sought to identify which short-term nGVS intensities were effective in patients who showed improvement with prolonged nGVS.

Methods: This trial registration was registered on August 10, 2018, (number: jRCT1080224083). Patients with peripheral vestibulopathy underwent closed-eye center-of-pressure (COP) measurements for 30 seconds without nGVS and with nGVS at 100-2000 μA to determine the optimal intensity (defined as the current yielding the greatest velocity improvement). Prolonged nGVS was then applied at this optimal intensity. Patients demonstrating at least a 10% improvement in mean velocity change during three hours of stimulation compared with placebo were classified as the “improvement” group, while others formed the “no-improvement” group. The percent change in COP velocity from baseline (no nGVS) at each intensity was compared between groups.

Results: The improvement group (n = 9) demonstrated significantly greater reductions in COP velocity than the no-improvement group (n = 30) at 100 μA and 1700 μA (*p* < 0.05). Additionally, compared with the no-improvement group, the improvement group exhibited enhanced responsiveness across a broader range of short-term nGVS current intensities.

Conclusion: In patients with peripheral vestibulopathy, the beneficial effect of prolonged nGVS on standing postural control is closely related to short-term responsiveness to nGVS across a wide range of current intensities. In particular, when the optimal stimulation intensity lies between 100 and 1700 μA, this relationship appears strongest.

## Introduction

A subset of patients with peripheral vestibulopathy remain resistant to vestibular rehabilitation, and their severe balance disorders are difficult to manage. Vestibular implants have been developed to enhance posture, gait, and quality of life in patients with bilateral vestibulopathy (BVP) [1]. However, there is a substantial risk of sensorineural hearing loss as a surgical complication [1]. Therefore, advances in minimally invasive treatments for refractory vestibular disease are needed.

Noisy galvanic vestibular stimulation (nGVS) delivers a zero-mean current noise signal to the vestibular system via electrodes placed bilaterally over the mastoid processes. Application of an optimal, imperceptible level of nGVS has been reported to improve standing postural stability and gait performance in both BVP patients and healthy individuals [2-9]. The rationale for nGVS is based on the concept of stochastic resonance [10,11], a phenomenon in which subthreshold signals in nonlinear systems are enhanced in the presence of an optimal level of noise. Several studies have demonstrated that nGVS can improve standing postural control for several hours after cessation of stimulation in both BVP patients and healthy subjects [12-15]. However, previous studies in BVP patients have primarily been open-label or single-blind trials comparing nGVS with no stimulation [4,5,7-9] and thus may not have adequately accounted for placebo effects or observer bias.

We recently conducted a multicenter, randomized, double-blind, placebo-controlled, crossover trial (jRCT1080224083; registered on August 10, 2018, in the Japan Pharmaceutical Information Center Clinical Trials Information registry) to evaluate the efficacy of prolonged nGVS in improving balance in patients with unilateral vestibulopathy (UVP) and BVP with severe postural instability refractory to vestibular rehabilitation. However, this trial did not demonstrate a significant improvement in postural stability after three hours of prolonged nGVS compared with placebo [16].

In that trial, the current intensity for prolonged nGVS was determined by first applying short-term nGVS at various current intensities to identify the level that produced the greatest improvement in balance performance. Despite using this individualized approach, the study did not show an overall benefit in postural stability. In the present study, we used data from the jRCT1080224083 trial to examine the relationship between the effects of short-term stimulation at various current intensities on postural control and the effects of prolonged stimulation at the optimal intensity performed on different days.

## Materials and methods

Study design and participants

In the present study, data obtained from the jRCT1080224083 trial were additionally analyzed [16]. The jRCT1080224083 trial was designed and conducted in accordance with the study protocol, the Declaration of Helsinki, and Japanese Good Clinical Practice. The protocol was approved by the Pharmaceuticals and Medical Devices Agency of Japan and by the institutional review board at each participating hospital. The trial was conducted at three centers in Japan: The University of Tokyo Hospital, The Tokyo Teishin Hospital, and Nagoya City University Hospital.

Inclusion criteria were as follows: (1) patients with UVP or BVP confirmed by ice water caloric testing (2 mL, 20 seconds); (2) patients with a total trajectory length (TTL) of the center-of-pressure (COP) of at least 180 cm over 60 seconds during posturography (Gravicorder G-620, Anima Inc., Tokyo, Japan) while standing with the eyes closed; (3) patients with imbalance lasting more than one year and persistent symptoms despite vestibular rehabilitation for over six months; (4) patients aged 20 to 85 years; and (5) patients who understood the study details and voluntarily provided written informed consent before participation.

Canal paresis was calculated as the difference between the maximal slow-phase eye velocity of each ear divided by the sum of the two velocities. UVP was defined as canal paresis of 20% or greater [17], whereas BVP was defined as a maximum slow-phase velocity of less than 10°/s bilaterally [18]. Because there is no established consensus on the definition of severe postural instability in posturography, we defined it based on the upper limit of posturographic findings in healthy elderly subjects. The TTL values in the closed-eye condition for 16 healthy participants (7 males, 9 females; mean ± SD = 113.2 ± 34.8 cm) aged 65-69 years were extracted from posturographic data used in our previous study [19]. The upper limit of normal (mean + 2 SD) was 182.8 cm. Therefore, in the present study, we defined severe postural instability as a TTL > 180 cm, which approximates the upper limit of normal for individuals aged 65-69 years.

Exclusion criteria were as follows: (1) presence of metallic implants such as cerebral artery clips, cochlear implants, or pacemakers (silver dental fillings were acceptable); (2) orthopedic problems, including fractures, sprains, or acute painful musculoskeletal conditions; (3) limb movement disorders due to cerebellar or spinal cord disease; (4) significant cardiac disease, including severe arrhythmias requiring a pacemaker (e.g., atrial fibrillation, severe QT prolongation, or second-degree or higher atrioventricular block) or severe heart failure impairing ambulation; (5) malignant tumors; (6) infectious diseases accompanied by fever, malaise, or dizziness; (7) pregnancy or postpartum status; (8) inability to walk independently; (9) skin abnormalities such as infections or wounds at the stimulation site, or a history of anaphylactic shock or severe allergy; (10) lack of or limited legal capacity; and (11) participation in another clinical trial within the three months preceding informed consent or planned concurrent participation in another clinical study.

Participants who provided written informed consent and met all inclusion and exclusion criteria were provisionally enrolled. These subjects underwent testing to determine their optimal nGVS intensity. Only subjects for whom an optimal intensity could be identified were subsequently enrolled in the main randomized, double-blind, placebo-controlled crossover trial. Subjects without an identifiable optimal intensity were excluded from the trial.

nGVS application

The nGVS was applied using electrodes placed bilaterally over the mastoid region and delivered by a portable stimulator (112 × 67 × 31 mm; 160 g including batteries) [4,20]. The stimulation waveforms were digitally stored and converted from digital to analog at 20 Hz. A zero-mean white noise galvanic vestibular stimulation signal ranging from 0.02 to 10 Hz was used. The current amplitude crossed 0 mA, with a peak-to-peak amplitude of 2 mA.

Posturography

In the posturography test, subjects performed two-legged stance tasks under eyes-closed conditions, both without nGVS and with nGVS, at a sampling frequency of 20 Hz. Three parameters were measured: velocity, area, and root mean square (RMS) of the COP movement in the XY plane. The mean velocity was calculated as the TTL divided by the measurement time. A fall was defined as any displacement of the foot during posturography testing, and this definition was standardized across all three centers.

Measurement of optimal intensity

The optimal intensity of nGVS was determined as follows: while standing on the posturography platform with eyes closed and without nGVS, the COP was measured three times for 30 seconds each, with two-minute intervals between trials. The average of these three measurements was used as the baseline value. Subsequently, nGVS was applied at current intensities of 100, 200, 300, 500, 700, 1000, 1200, 1500, 1700, and 2000 μA, with two-minute intervals between each condition. COP was measured for 30 seconds under eyes-closed conditions at each intensity. Patients were considered to have an optimal intensity if both of the following criteria were met: (1) Multiple consecutive current intensities between 100 and 1000 μA were imperceptible and showed improved velocity compared with baseline. (2) The current intensity producing the greatest improvement in velocity demonstrated more than a 10% improvement over baseline. Patients who met these criteria were enrolled in the main study. The optimal intensity used for enrollment was defined as the current intensity that produced the largest improvement in velocity among the above range. Because the safety of prolonged high-current stimulation for four hours was uncertain, the maximum current used in the main clinical trial was limited to 1000 μA.

Randomization and masking

All personnel involved in the main trial, except those responsible for randomization of the investigational device, were blinded to the allocation information. Subjects enrolled in the main trial were assigned to two groups (Group A or Group B) in a 1:1 ratio using block randomization (block size of 4) via a web-based randomization system (Datatrak One). Randomization was stratified by type of vestibulopathy (UVP or BVP) and by TTL during the screening period (≥200 cm or <200 cm). In Group A, the effect of nGVS at the optimal intensity on body balance was evaluated in “Session I,” and the placebo effect (0 µA) was evaluated 14 days later (with a minimum interval of 7 days) in “Session II.” In Group B, the placebo effect was evaluated in “Session I,” followed 14 days later (with a minimum interval of 7 days) by assessment of nGVS at the optimal intensity in “Session II.” Masking methods were described in detail in our previous report [16].

Procedures of the main randomized, double-blind, placebo-controlled crossover trial

Subjects enrolled in the main trial visited the hospital 14 days (minimum 7 days) after enrollment and were randomly assigned to either Group A or Group B in a 1:1 ratio. In both Session I and Session II, subjects received either nGVS at the optimal intensity or placebo stimulation for four hours, followed by observation for an additional three hours after stimulation ended. During each session, subjects remained in the hospital where the trial was conducted but were not required to perform any specific tasks other than the examinations outlined in the study protocol. The washout period between sessions was 14 days (minimum 7 days). In Group A, Session I involved nGVS and Session II involved placebo stimulation, while Group B underwent the reverse order. Eyes-closed posturography was performed immediately before stimulation, immediately after the start of stimulation, 0.5 hours later, and then hourly from 1 to 7 hours post-stimulation. The baseline value was defined as the average of three measurements taken at two-minute intervals immediately before stimulation.

Statistical analysis

The sample size was predetermined based on the jRCT1080224083 trial [16]. The primary outcome in that trial was the percent change from baseline in the velocity of the COP at all measurement time points from the start of stimulation to three hours [16]. The mean percent change during the nGVS period was compared with that during the placebo period.

When calculating the sample size for jRCT1080224083, a 10% difference in the percent change from baseline in COP velocity between the nGVS and placebo periods was considered clinically meaningful, based on previously reported differences between patients with vestibulopathy and healthy controls [21]. In addition, according to our earlier study, the minimally important difference (MID) for subjective improvement in velocity after nGVS treatment in patients with BVP was estimated to be -0.43 cm/s using the anchor-based method [22]. In jRCT1080224083, the baseline mean velocity during the nGVS period was 4.44 cm/s, and that during the placebo period was 4.53 cm/s. These baseline velocities correspond to approximately 10% of the absolute value of the MID. Accordingly, patients who showed at least a 10% improvement in the primary outcome were classified as the “improvement group,” while those who did not meet this criterion were classified as the “no-improvement group.” Patient characteristics, including age, height, weight, body mass index, TTL, optimal intensity, sex, sensory threshold, TTL during the screening period (>200 cm or <200 cm), and type of vestibulopathy (BVP or UVP), were compared between groups using the Wilcoxon rank-sum test or Fisher’s exact test.

To determine the optimal intensity of nGVS, COP was measured for 30 seconds under eyes-closed conditions without nGVS (baseline) and with nGVS at 100, 200, 300, 500, 700, 1000, 1200, 1500, 1700, and 2000 μA. At each current intensity, the percent improvement in velocity from baseline was compared between the improvement and no-improvement groups using the Wilcoxon rank-sum test. Statistical analyses for jRCT1080224083 and the present study were performed using SAS version 9.4 (SAS Institute Inc., Cary, NC, USA) and EZR version 1.55 [23], respectively. A p-value < 0.05 was considered statistically significant.

## Results

**Figure 1:**
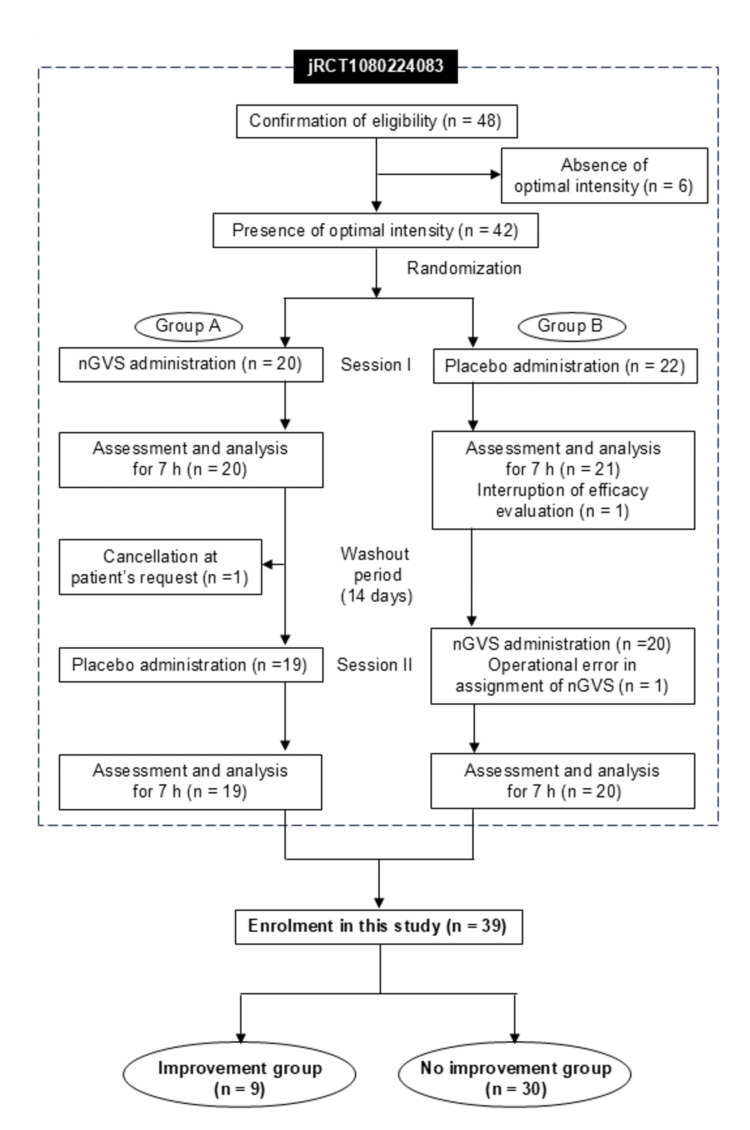
Study profile. nGVS: noisy galvanic vestibular stimulation.

A total of 42 patients were enrolled in jRCT1080224083. One patient perceived stimulation during Session I, compromising blinding, and was therefore excluded from the efficacy evaluation. Another patient declined to participate in Session II, and one patient received placebo stimulation in both sessions due to an operational error. These three patients were excluded, leaving 39 patients (26 males and 13 females; mean age ± SD, 68.1 ± 10.5 years) for analysis (Figure 1). Among these, 9 patients were classified into the improvement group and 30 into the no-improvement group. Table 1 summarizes the characteristics of both groups, showing no significant differences in any patient-related parameters between them.

**Table 1:** Characteristics of enrolled patients. Data are presented as mean ± SD or number and percentage, as appropriate. Test statistics (W) and p-values indicate comparisons between the improvement and no-improvement groups, calculated using the Wilcoxon rank-sum test^a^ or Fisher's exact test^b^, as appropriate. Statistical significance was defined as p < 0.05. TTL: total trajectory length, BVP: bilateral vestibulopathy, UVP: unilateral vestibulopathy.

	Total	Improvement	No improvement	W	p-value
	(n = 42)	(n = 12)	(n = 30)		
Age (years) (mean, SD)	68.1 (10.5)	64.8 (9.9)	69.0 (10.7)	99	0.236^a^
Height (cm) (mean, SD)	163.73 (8.25)	167.79 (9.44)	162.51 (7.61)	169	0.264^a^
Weight (kg) (mean, SD)	66.46 (10.98)	69.46 (8.32)	65.56 (11.62)	161	0.395^a^
Body mass index (kg/m^2^) (mean, SD)	24.70 (3.21)	24.76 (3.38)	24.68 (3.22)	131.5	0.920^a^
TTL (cm) (mean, SD)	328.48 (114.73)	319.60 (82.71)	331.14 (123.81)	135	1.000^a^
Optimal intensity (µA) (mean, SD)	482.1 (270.4)	477.78 (185.59)	483.3 (293.7)	148	0.671^a^
Sex (N, %)					
Female	13 (33.3 %)	1 (11.1 %)	12 (40.0 %)		0.225^b^
Male	26 (66.7 %)	8 (88.9 %)	18 (60.0 %)		
Presence of sensory thresholds (N, %)					
Yes	27 (69.2 %)	8 (88.9 %)	19 (63.3 %)		0.228^b^
No	12 (30.8 %)	1 (11.1 %)	11 (36.7 %)		
TTL at screening period (N, %)					
≥200 cm	36 (92.3 %)	9 (100.0 %)	27 (90.0 %)		1.000^b^
<200 cm	3 (7.7 %)	0 (0.0 %)	3 (10.0 %)		
BVP or UVP (N, %)					
BVP	14 (35.9 %)	5 (55.6 %)	9 (30.0 %)		0.238^b^
UVP	25 (64.1 %)	4 (44.4 %)	21 (70.0 %)		

**Figure 2:**
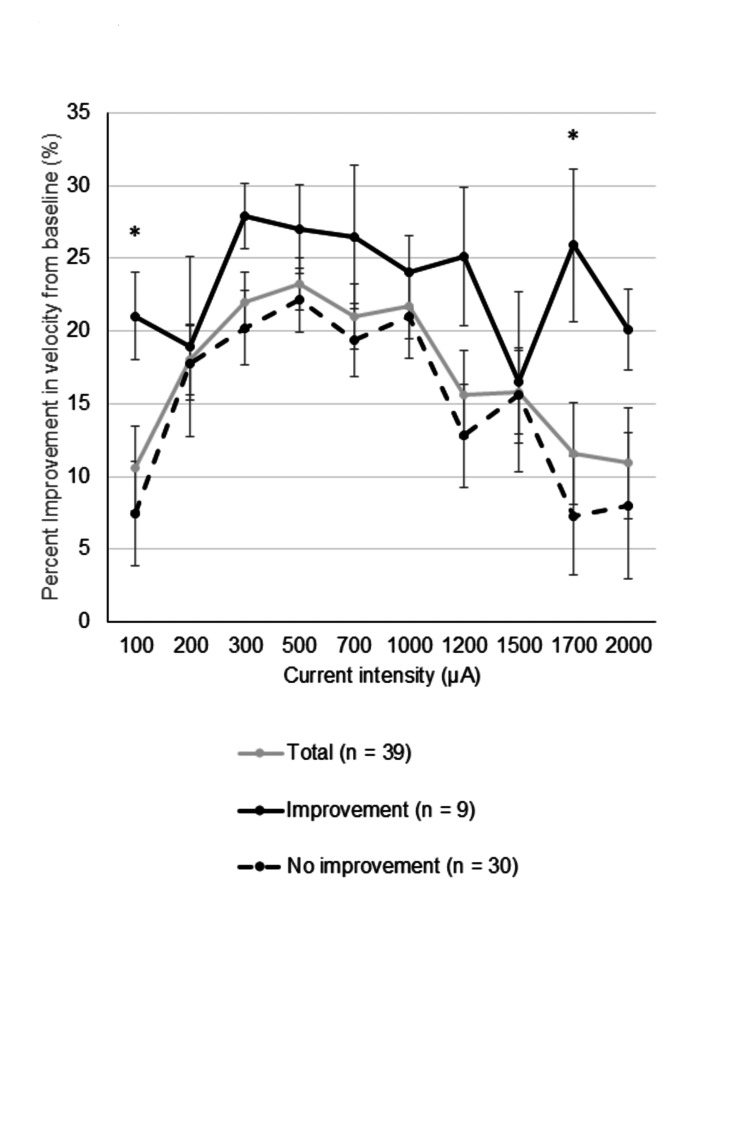
Percent improvement in velocity from baseline during 30 seconds of short stimulation at each current intensity. The improvement group had a significantly greater improvement than the no-improvement group at 100 µA and 1700 µA. Data are presented as mean ± SE. *p < 0.05 was considered statistically significant (Wilcoxon rank-sum test).

Figure 2 shows the percent improvement in velocity from baseline during 30 seconds of short stimulation at each current intensity. Both groups showed improvement across all current intensities, but the improvement group exhibited significantly greater reductions in COP velocity than the no-improvement group at 100 µA and 1700 µA (p = 0.039 for both, Wilcoxon rank-sum test). The improvement group tended to show enhancement over a wider range of current intensities, whereas the no-improvement group showed less improvement as the current deviated from 500 µA. These findings suggest that the beneficial effect of prolonged nGVS on standing postural control is related to short-term nGVS effects observed over a broader range of current intensities.

## Discussion

The current intensity used in long-duration nGVS is typically determined by applying nGVS at various current intensities for short periods and selecting the level that produces the greatest improvement in body balance. In our previous study, however, we found that the optimal intensity varied from day to day [24]. Therefore, one possible explanation for the differing results between previous studies showing the efficacy of nGVS and the jRCT1080224083 trial, which did not, is that the current intensity used in the main trial of jRCT1080224083 may not have been optimal, as it was conducted two weeks after determining the optimal intensity. Nevertheless, in clinical practice, it is difficult to determine the optimal intensity each time nGVS is applied.

The present analysis used data from jRCT1080224083 [16]. The previous randomized controlled trial evaluated the efficacy of prolonged nGVS in patients with vestibulopathy but found no significant improvement in postural stability. In contrast, this study aimed to investigate whether the short-term effects of nGVS at various current intensities are associated with the effects of prolonged nGVS. This secondary, exploratory analysis provides additional insight into potential responders, which were not identified in the previous publication [16]. Our findings suggest that patients whose postural control improved with prolonged nGVS also exhibited improvement with short-term nGVS over a broader range of current intensities. Although the optimal intensity for balance improvement may vary from day to day, patients who respond to a wider range of current intensities may be less affected by such variability.

The reason why the ameliorating effects of prolonged nGVS on postural control are associated with those of short-term nGVS over a broader range of current intensities remains unclear. Previous studies have reported intra- and inter-individual variations in skin and subcutaneous tissue impedance, and that the electrical properties of the skin can change significantly when an iontophoresis current is applied [25]. It is possible that the condition of the skin and subcutaneous tissues at the electrode site influenced the results of this study. Patients who exhibit improvement with short-term nGVS across a broader range of current intensities may be less affected by such changes in the electrical properties of the skin.

Previous studies have highlighted that the emergence and strength of stochastic resonance (SR) in neural networks depend on network topology, including modular organization, small-world connectivity, and the presence of multiple pathways [26]. These factors can enhance signal transmission and detection, providing a theoretical framework for understanding variability in responses to weak stimuli. However, most existing studies have focused primarily on the amplitude of SR responses, and the mechanisms underlying individual differences in the effective noise range for SR remain unclear. Further research is needed to clarify why some individuals respond effectively across a broader range of stimulation intensities.

This study has several limitations. First, we did not control for potential learning effects during the procedure for determining the optimal intensity, which involved repeated short stimulations for 30 seconds at various current intensities. Second, we did not assess parameters related to the asymmetry of postural COP displacement. The velocity parameter used in this study may therefore be nonspecific to the expected therapeutic effects of nGVS on vestibulopathy. Third, vestibular stimulation by nGVS may be influenced by the condition of the skin and subcutaneous tissues at the electrode site, as well as by anatomical differences in the mastoid region. However, it is difficult to quantify the extent to which transcutaneous currents actually stimulate the vestibular system. Finally, the relatively small sample size may have limited the statistical power and generalizability of our findings.

## Conclusions

The optimal current intensity for prolonged nGVS is typically determined by short-term application of various current intensities and selecting the intensity that maximally improves postural control. In patients with peripheral vestibulopathy, the improvement in standing postural control following prolonged nGVS is closely associated with the responsiveness to short-term nGVS across a broad range of current intensities. Notably, the strength of this association is greatest when the optimal stimulation intensity falls within the 100-1700 µA range.
